# Incidence rate and time to serious adverse events among rifampicin resistant tuberculosis patients in Georgia treated with new and repurposed anti-tuberculosis drugs, 2016–2018

**DOI:** 10.4081/monaldi.2021.1649

**Published:** 2021-01-14

**Authors:** Mariana Buziashvili, Hayk Davtyan, Yuliia Sereda, Olga Denisiuk, Ogtay Gozalov, Nino Lomtadze, Arax Hovhannesyan

**Affiliations:** 1 Scientific Research Unit, National Center for Tuberculosis and Lung Diseases, Tbilisi, Georgia; 2 Tuberculosis Research and Prevention Center, Yerevan, Armenia; 3 World Health Organization, Regional Office for Europe, Copenhagen, Denmark; 4 Alliance for Public Health, Kyiv, Ukraine

**Keywords:** Tuberculosis, drug resistance, new/repurposed anti-TB drugs, SAEs

## Abstract

Considering the complexity of second-line anti-tuberculosis (TB) treatment regimens, the management of drug-resistant TB (DR-TB) in Georgia remains a major challenge. Since the introduction of new and repurposed anti-TB medications, the implementation of active TB Drug Safety Monitoring (aDSM) was a critical program component to help establish safety and manage all treatment related Serious Adverse Events (SAEs). In our study, we aimed to describe the occurrence, characteristics and timing of SAE among patients with Rifampicin Resistant and Multi-Drug Resistant TB (RR/MDR-TB) receiving new and/or repurposed anti-TB medications (bedaquiline, delamanid, linezolid, clofazimine, imipenem) during the period of 2016–2018 in Georgia and identify predictors of SAE. The data were obtained from the medical charts, electronic database and standardized aDSM reports During 2016–2018 period in total 970 people with RR/MDR-TB were notified in Georgia and 388 of them received new and/or repurposed TB drugs as part of their treatment regimen and all were included into the study. The results showed a total of 73 SAEs registered among 49 (12.6%) patients receiving new and/or repurposed drugs. The overall SAE incidence rate per 100 person-months was 1.16. The severity of the majority of the SAEs (46.6%) was grade III and 21.9% were grade IV. The most common SAE reported was hepatotoxicity, with an incidence of 0.26 per 100 person-month (n=16, 21.9%) followed by cardiotoxicity with an incidence of 0.16 per 100 person-month (n=10, 13.7%). Median time to SAE occurrence was 183 days (IQR 84 – 334) after treatment initiation. Resistance profile was the only predictor associated with occurrence of a SAEs. There was increased hazard of SAEs among patients with XDR-TB (adjusted HR=2.18, 95% CI: 1.12–4.23). Our findings on SAEs among patients treated with new or repurposed anti-TB drugs are echoing the findings available in the literature. They highlight the need for close monitoring of patients and underlines the importance of the aDSM during the whole treatment. Safety profile of the medications and combinations used are yet to be established and larger datasets comprised of patients receiving same treatment regimens need to be utilized.

## Introduction

Worldwide, around 10 million people fall ill with tuberculosis (TB) each year. It is one of the top 10 causes of death globally, and the leading cause of death from the single infectious agent (*Mycobacterium tuberculosis*) [1]. Moreover, the burden of drug-resistant TB (DR-TB) is of major concern at global, regional and country levels. In 2018, there were approximately half a million (range: 417,000 – 556,000) new cases of rifampicin-resistant TB (RR-TB), 78% of which had multidrug-resistant TB (MDR-TB) [1]. Globally, 3.4% of new TB cases and 18% of previously treated cases had MDR/RR-TB, with the highest proportions located in the countries of the former Soviet Union [1]. The definitions of relevant terminology are provided in the Supplementary Table 1.

As of 2018, the estimated TB incidence rate in Georgia was 80 per 100,000 population, while the RR/DR-TB incidence reached 14 per 100,000 population and the proportion of RR/DR-TB among new and previously treated TB cases was 10.5% and 35.5%, respectively. Extensively Drug-Resistant (XDR)-TB accounted for 17.9% of all MDR-TB cases [2]. The treatment success rate among RR-TB patients enrolled into second-line TB treatment in Georgia is showing increasing trends, reaching 65,7% in 2016 cohort, although still remaining low compared to the Regional target of 75% [2].

Compared to the standard 6-month treatment for drug-susceptible TB, treating drug-resistant TB is relatively difficult and complex, as the treatment is expensive and lengthy, requiring the use of second-line anti-TB drugs (SLDs) for up to 24 months, with treatment outcomes remaining suboptimal in many cases due to drug-related adverse events [3–5]. Toxicity caused by SLDs and long treatment were often reported as the main reasons compromising treatment adherence and leading to early treatment discontinuation and high prevalence of unfavorable treatment outcomes [5,6]. Since 2015, Georgia introduced new and repurposed anti-TB medications for the management of difficult-to-treat TB patients including bedaquiline (Bdq), delamanid (Dlm), linezolid (Lzd) clofazimine (Cfz) and imipenem (Imp). Because TB treatment requires combined administration of drugs with proved effectiveness, the introduction of new and repurposed drugs was a good opportunity for the National Tuberculosis Programme (NTP) in Georgia to improve the RR-TB treatment outcome, especially of those with high drug-resistance profile. However, as the newly introduced medications, especially Bdq and Dlm, were approved with limited human data, active TB Drug Safety Monitoring (aDSM) was seen as an important program component to help establish safety [7].

Following the programmatic implementation of new and repurposed drugs in the country, an aDSM and a management framework were elaborated to monitor, manage and minimize all treatment related Serious Adverse Events (SAEs). An aDSM assumes active and systematic clinical and laboratory assessment of patients on treatment with any anti-TB medicines and plays a vital role for new TB medicines, or novel MDR-TB or XDR-TB regimens, to detect, manage and report suspected or confirmed drug toxicities [7,8]. While all detected AEs need to be managed, the core package of aDSM requires that as a minimum, all SAEs occurring should be documented and reported in patients monitored.

There are several publications related to evaluation of the safety and effectiveness of new and repurposed anti-TB drugs from clinical trials around the world [9]. However, drug safety information from the real programmatic sources are still limited, as those treatment regimens were introduced in the countries recently. To address this gap, our study aimed to describe the occurrence, characteristics and timing of SAEs among the people with RR/MDR-TB receiving new and repurposed medicine and identify predictors of SAEs.

## Methods

### Study design

A cohort study of RR-TB patients initiating treatment with new and repurposed anti-TB medications in the country of Georgia, using secondary data from patient medical charts, electronic database and standardized aDSM reports during 2016–2018, inclusive.

### Study setting – general, study site and study period

There are total of 67 TB provider facilities serving for the NTP aims in Georgia, 10 of them representing central facilities for each region of the country, including the main facility, the National Center for Tuberculosis and Lung Diseases (NCTLD) in the capital, Tbilisi.

Every person with signs and symptom corresponding with TB is examined clinically and radiologically along with laboratory tests, including rapid molecular tests for detection of *M. tuberculosis* and rifampicin resistance (Xpert MTB/RIF test, first line LPA and second line LPA), smear microscopy, cultural tests and phenotypic drug susceptibility tests (DST) [10]. Depending on sputum smear results, as well as patient clinical condition, the anti-TB treatment is initiated in either inpatient, or outpatient departments of TB provider facilities. The central DR-TB Committee (DRC), based at the NCTLD, is the only authorized entity countrywide to assign appropriate DR-TB treatment and/or take clinical decisions in case of treatment complications. The process is regulated through weekly video-based ECHO (Extension for Community Healthcare Outcomes) committees, allowing all TB facilities countrywide to attend the DRC simultaneously and limiting the need of patient to travel to any specific facility for treatment initiation or modification [11]. Treatment monitoring over the course of DR-TB treatment is implemented in-line with WHO recommended treatment monitoring plan and national guideline, outlining the frequency of relevant laboratory and instrumental tests, as well as chest X-ray and other necessary analysis [7,12].

While inpatient, all TB patients are evaluated by their physicians for clinical signs and symptoms of any adverse events on a daily basis, and laboratory examinations are scheduled for every month, irrespective of inpatient or outpatient treatment. All health care providers involved in the management of DR-TB are trained in identification, recording, reporting and management anti-TB treatment related adverse events. If any abnormality is detected, a patient is monitored with relevant laboratory tests more closely, while simultaneously assessing causal relationship with anti-TB medications. The DRC then decides what actions are to be taken towards management of specific adverse events. The evaluation of adverse events is based on the Division of Acquired Immunodeficiency Syndrome (DAIDS) table for grading the severity of adult and pediatric adverse events [13]. All SAEs are recorded on a standardized SAE reporting form and reported to NCTLD’s Pharmacovigilance (PV) Unit, while other adverse events are just recorded into patient medical charts.

### Study population

All RR-TB patients receiving any of the new and/or repurposed drugs of interest (Bdq, Dlm, Lzd, Cfz, Imp) in any TB provider facility in the country of Georgia during the period of January 2016 through December 2018 were included into current analysis. Patients were eligible to receive new or repurposed drugs in their treatment regimen if they had pre-extensively drug-resistant (pre-XDR) or extensively drug-resistant TB (XDR-TB), intolerance to fluoroquinolones or second-line injectable drugs, drug-resistance and/or intolerance to two or more of the following medications: ethionamide, cycloserine or para-aminosalicylic acid (PAS). Patients with severe disease and bad clinical conditions were also eligible to receive new and repurposed drugs. The treatment regimens, including injectable anti-TB medications, were designed based on patient DST profile, medical history, clinical condition and ongoing disease severity.

### Data source

The routine data collection in the NTP is conducted electronically through the national TB database. Information on patient demographic, socio-economic status and medical history are collected at the baseline, along with TB history and treatment-related baseline and follow-up clinical evaluations (mycobacteriological and biochemical analysis, chest radiography, *etc*.).

All SAEs identified over the course of treatment were prospectively collected, using a standardized SAE reporting form and reported to PV Unit at the NCTLD. The SAE reporting form includes variables on patient demographic and medical information, and detailed information on SAEs. The SAEs are evaluated using standardized seriousness criteria of death, life-threatening, hospitalization required/prolonged, persistent or significant disability/incapacity, congenital anomaly/birth defect and otherwise medically important, as well as by seriousness grades from 1 through 4, based on the DAIDS table for grading the severity.

The existing standardized SAE reporting forms were used to extract information on serious adverse events and national electronic TB database was used for patient demographic and medical history information.

### Variables

The main outcomes of interest of the study are the SAEs and time to SAE development. Explanatory variables considered were as follows: age, sex, resistance profile, treatment history, co-morbidities: diabetes, Human Immunodeficiency Virus (HIV), Hepatitis C Virus (HCV), alcohol use, tobacco use, illicit drug use and treatment outcome. We also collected the detailed characteristics of SAE, including, SAE type, SAE grade, SAE outcome, actions taken in regard to the SAE, suspected medication for the SAE, as well as dates of treatment initiation, date of SAE occurrence, resolution and date of treatment outcome.

Diabetes status was identified based on clinical history or blood glucose measurement followed by clinical evaluation of an endocrinologist. All patients were offered a test for HIV and HCV at the baseline and the confirmation for positive rapid tests results were received from the National Center for Disease Control and Public Health (NCDC&PH) laboratory. Data on smoking status, alcohol and illicit drug use were obtained during the baseline evaluation from patient self-reports. For the current analysis, we classified drug resistance pattern into 3 categories: rifampicin resistant, pre-XDR and XDR.

### Statistical analysis

Data on all patients initiating treatment with new and repurposed anti-TB medications during 2016–2018 were filtered and exported and merged with the aDSM database using one-by-multiple approach accounting the possibility of one patient having several SAEs. Descriptive statistics was used to describe patient demographic and clinical characteristics. The categorical variables were described using percentages and continuous variables – using mean and standard deviations (SD), or median and interquartile range (IQR).

To compute SAE incidence rate, overall patient follow-up time was calculated which is defined as a sum of total duration from treatment start, up to treatment outcome, including death, loss to follow-up or end of the study. The SAE incidence rate was expressed as total number of SAEs over the overall follow-up time per 100 person-months and plotted.

The frequency of SAEs was presented by type, outcome, actions taken in regard to the SAE and a suspected medication. For each type of SAEs the rate, median time to development with IQR and a total range were calculated.

To assess the predictors of SAEs hazard ratio as a measure of association between predictors and outcome of interest was computed. The patients who died or were lost to follow-up before the end of treatment were censored. A univariable analysis was performed to assess factors associated with SAE occurrence, using an extended Cox regression (The Prentice, Williams, and Peterson Total Time Model) to account for recurrent events. Sample size allowed to include all factors into the multivariable analysis disregarding their significance level except for the intravenous drug use. Adjusted hazard ratio, confidence interval and p-value were calculated and presented in the final model. Validity assumption of proportionality was assessed using global test of proportionality using Schoenfeld residuals. Plot to show mean cumulative frequency of SAE disaggregated by the type of resistance among DR-TB patients was constructed. Analysis was done using R, version 3.5.2 software (©R Foundation for Statistical Computing, 2016) [14].

## Results

### Cohort description

Out of 970 persons notified with RR/MDR-TB during 2016–2018, 388 received new and/or repurposed anti-TB drugs as part of their treatment regimen and all were included in the study. Median follow-up duration was 19.1 months (IQR: 12.1–20.1 months, min-max: 0.3–27.1 months). The average (SD) age of patients was 39.6 (13.1). Majority of the patients were male (n=304, 78.3%), and 174 (44.9%) were new cases. Twenty-six (6.7%) were co-infected with HIV, while the HIV status was unknown for 29 patients (7.5%). Fifty-seven (14.7%) had positive HCV test results and in 163 patients (42%) HCV was not recorded. Diabetes was present in 33 patients (8.5%). Proportions of patients who use tobacco, alcohol and intravenous drug were 53.1% (n=206), 40.2% (n=156) and 5.4% (n=21) respectively.

Among the 388 patients included, 269 (69.3%) were treated with bedaquiline, 200 (51.5%) with delamanid, 81 (20.9%) with both bedaquiline and delamanid, 255 (65.7%) with linezolid and 217 (55.9%) with clofazimine. The proportion of patients with respect to the resistance types were as follows: RR – 1% (n=4), MDR – 38.4% (n=149), pre-XDR – 49.7% (n=193) and XDR – 10.8% (n=42). Treatment success (cured or treatment completed) was registered in 244 patients (62.9%), unsuccessful outcome was present in 78 patients (20.1%) and for the rest (n=66, 17%) the treatment outcome was not evaluated. More detailed characteristics of the study population is presented in the Supplementary Table 2.

### Characteristics of serious adverse events

**Figure 1.**
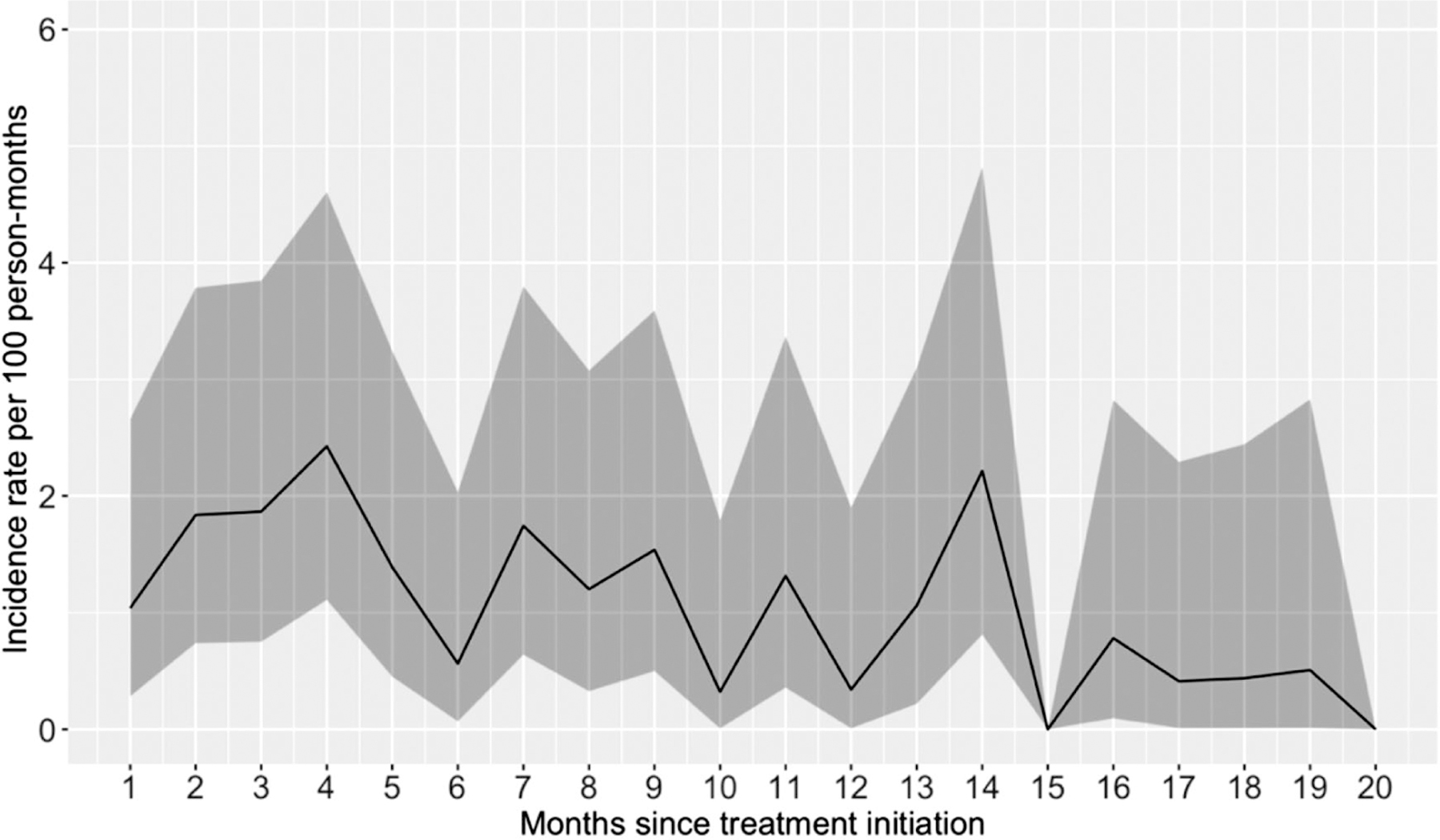
SAE incidence rate among DR-TB patients in Georgia that initiated treatment between 2016 and 2018 (N=388). Grey ribbon indicates 95% confidence interval; incidence rate was estimated for monthly time intervals, for example the rate at 2 months means the period above 1 month and less or equal 2 months.

In total 73 SAEs were registered in 49 patients (12.6%, 95% CI: 9.5%–16.4%), where 31 (8.0%) had one SAE, 13 (3.4%) had two SAEs, four (1.0%) patients had 3 SAEs, and one patient (0.3%) – 4 SAEs. Overall rate of SAEs per 100 person-months was 1.16. SAE occurrence is presented in Figure 1. There was no clear trend and we found that SAE could occur any time during the treatment.

The majority (46.6%) of the SAEs were of grade III in terms of severity, while 16 (21.9%) were of grade IV. The most common SAE type was hepatotoxicity, with an incidence of 0.26 per 100 person-month (n=16, 21.9%), followed by cardiotoxicity with an incidence of 0.16 per 100 person-month (n=10, 13.7%). Out of 49 patients with SAEs, there were 9 patients (18.4%) with QT prolongation, requiring drug interruption in seven instances and permanent discontinuation in one instance (Table 1; Supplementary Table 1).

By the onset of occurrence neurotoxicity was the earliest SAE with median time of 110 days (IQR 69–325). In general, median time to SAE occurrence was 183 days (IQR 84 – 334) after treatment initiation and ranged from 5 to 700 days.

The most frequent culpable agent was clofazimine, which was suspected to be the cause for 21 (28.8%) SAEs followed by delamanid (n=15, 20.5%), cycloserine and linezolid (in both cases n=13, 17.8%). In 39 (53.4%) cases of SAEs the suspect medication was interrupted and in three (4.1%) cases medication was permanently withdrawn. Majority of SAEs resolved without sequelae (n=44, 60.3%), 6 (8.2%) resolved with sequelae, 13 (17.8%) were not resolved, 7 (9.6%) were fatal and the remaining 3 (4.1%) were resolving at the time of data collection (Table 2).

### Factors associated with the occurrence of SAEs

**Figure 2.**
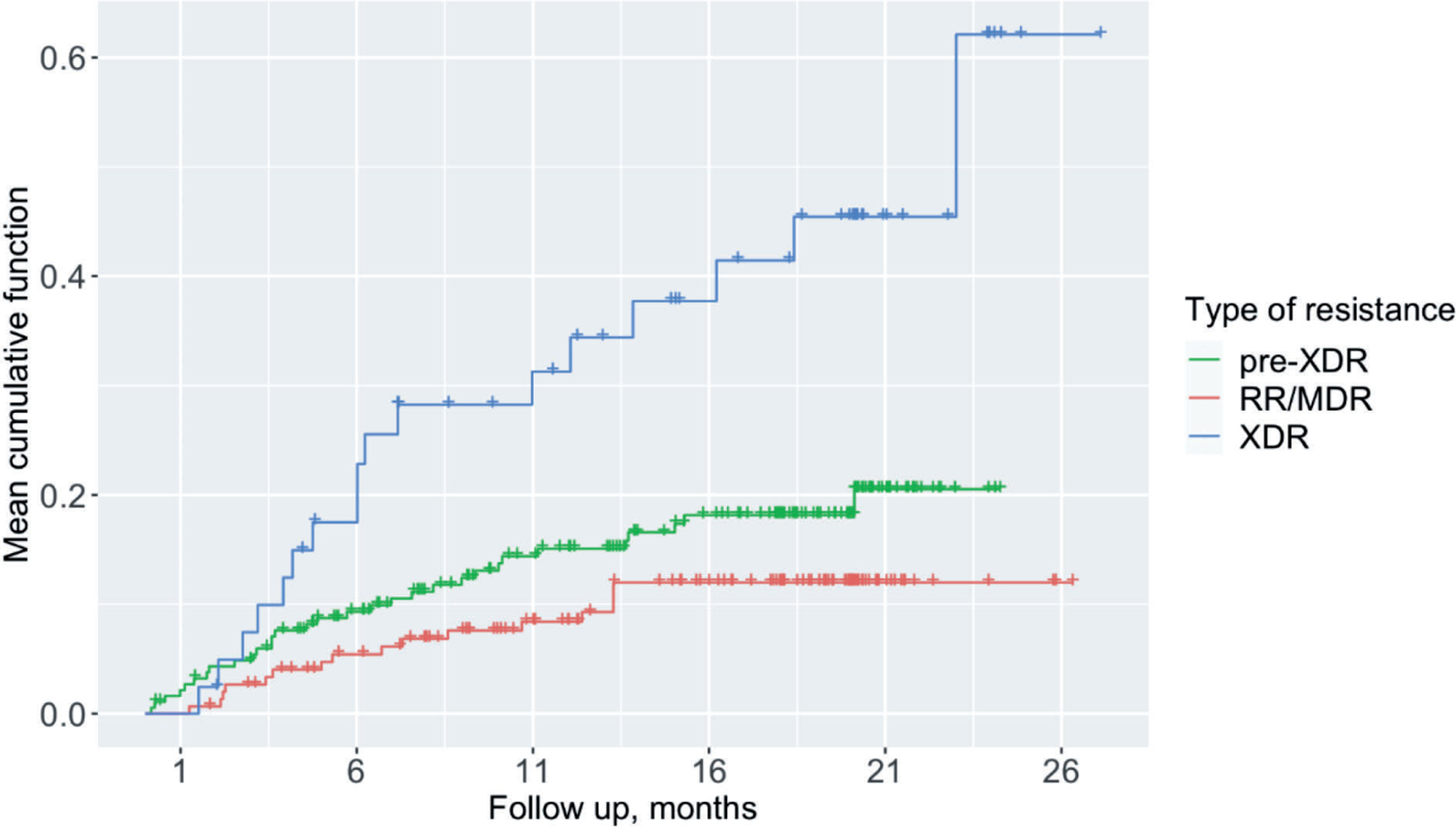
Mean cumulative frequency of SAE disaggregated by the type of resistance among DR-TB patients in Georgia that initiated treatment between 2016 and 2018 (N=388).

Drug-resistance profile was the only characteristic associated with the occurrence of SAEs in a stepwise manner. The highest hazard of SAE was observed among those with XDR-TB, followed by pre-XDR-TB (Figure 2).

In multivariable analysis, after adjusting for all covariates, patients with XDR-TB had over 2 times higher likelihood of development of SAE compared to those with RR/MDR-TB (adjusted HR=2.18, 95% CI: 1.12–4.23, p=0.021). Results of the adjusted and unadjusted analyses are presented in Table 3.

### Description of SAEs with fatal outcome

Among 7 SAEs with fatal outcome, only in 4 cases the causal relationship with anti-TB treatment was assessed as possible (Supplementary Table 3). Cycloserine was deemed as the responsible agent in two deaths which occurred as a result of suicide. One of those patients had a history of resolved depression and anxiety. The second patient had a history of psychotropic drug abuse. Both patients were HCV co-infected.

The third patient developed kidney injury, which progressed to decrease in level of potassium, increased creatinine, QT prolongation and renal insufficiency. Causal relationship with Cfz and moxifloxacin (Mfx) was established as possible.

The fourth patient had a history of alcohol and drug abuse, which could have potentially worsened the tolerance of anti-TB medication. Cfz and Dlm were recognized as possible causal agents for this patient who had extremely low BMI at baseline (14.4) as well as with symptoms of constant nausea, vomiting and fatigue.

The remaining three patients with fatal outcome had severe comorbidity of cardiovascular disease, COPD and cancer, respectively and causal relationship between the SAE and TB treatment was not established.

## Discussion

Our study demonstrated that use of new and repurposed drugs in RR/MDR-TB treatment regimen in general was well tolerated. Of all patients exposed to new and repurposed drugs, 12.6% (95% CI: 9.5%−16.4%) experienced SAE. This is comparable to earlier reports: the first cohort of difficult-to-treat RR/MDR TB patients in Armenia and Georgia that received new and repurposed drugs as a part of compassionate use between 2013–2015 showed 17.1% of SAE. However, the proportion of the XDR-TB patients in that cohort was higher compared to the current study [15,16]. Our findings are similar to those observed in the Global aDSM project report comprised of 26 countries and showing 11.3% of SAE in RR/MDR cohorts treated with regimens containing novel and repurposed drugs [9]. It should be noted that global aDSM project includes only drug-related AEs, while we report all SAE, regardless of their association with the drugs in the treatment regimen. Therefore, our data compared to global aDSM over-estimate the risk of SAE.

In general, there is a large variation in the frequency of SAE across different studies, which could be attributed to the differences in practices, capacities of health services as well as robustness of country-specific aDSM systems to identify and report AE. It is possible that there may be various levels of bias introduced in different studies due to the differences in the skills of the healthcare workers responsible for detecting and reporting adverse events. The frequency of SAEs among MDR-TB patients receiving standard treatment regimen according to 2011 RR/MDR-TB treatment guidelines ranged from 6% to 43% [17–19]. Thus, observed occurance of SAEs in our study population migh be considered as reasonably low compared to pre-2016 WHO-reccomended regimens for drug-resistant TB.

While many studies demonstrated that AEs are more frequent at the beginning of the treatment [20] and the likelihood of occurrence of AEs gradually declines [21]. In our study, there was no clear trend in SAE occurrence over time: SAEs could occur at any time point of the treatment.

Clofazimine and delamanid were found to be the first and second most commonly suspected anti-TB medication causing SAE, while in a multi-centered prospective study evaluating frequency and severity of AEs in patients receiving new and repurposed TB drugs linezolid was considered to cause the highest proportion of SAEs [9]. However, these differences could be partially explained by the differences of the treatment regimens.

Having an XDR-TB was significantly associated with increased likelihood of SAE occurrence (adjusted HR=2.18, 95% CI:1.12–4.23). This is an expected result as XDR patients are receiving more aggressive treatment compared to other patients, thus are prone to have more AEs.

Of special concern is that there were two cases of suicide both associated with the use of cycloserine. It is well established that cycloserine might trigger depression, psychosis and suicidal thoughts in patients with RR/MDR-TB. According the available systematic review, absolute risk of SAE due to cycloserine among patients on longer MDR-TB regimen is 7.8% [22]. However, SAEs with fatal outcomes are rarely observed [23]. We speculate that psychiatric vulnerability is compounded by pre-existing depressive state of RR/MD-TB patients, attributed by social stigma, weak family and community support. This is especially true for Georgia, where psychiatric symptoms are culturally taboo. Earlier studies in other settings demonstrated that prevalence of the depression among RR/MDR-TB patients could be as high as 42% and mental well-being has a potential to improve TB outcomes [24,25]. The low number of reported SAEs related to psychiatric disorders suggests that patient’s mental health disorders in TB control in Georgia are most likely undercounted. The observed 62.8% treatment success rate is comparable to average national [2].

The strength of our study is that nationwide data over three years were analyzed and results represent all patients that started TB treatment with new and repurposed medicines between 2016–2018. Initial data was collected with the use of a standardized and self-explanatory forms, which decreases likelihood of errors and reporting bias of collected data. One of the limitations of the study is that the data on treatment regimens changes were not available and the percentages of patients using specific medications throughout the treatment were not possible to derive. Our system was designed to collect only SAE, which present only small proportion of total number of AE and does not provide comprehensive pattern of all drug toxicity related issues experienced by patients on RR/MDR-TB treatment regimen. Additionally, we did not have control over the errors that may be present in the initial dataset, compiled over three years by TB physicians working across the country. This risk was somewhat mitigated by using standardized forms for the data collection.

## Conclusions

Nearly one in ten patients that are being treated with new and/or repurposed anti-TB drugs develop at least one SAE which may occur at any month of the treatment. Severe drug-resistance profile was a major factor associated with SAEs. This finding indicates the need for the close monitoring of the patients during the whole course of treatment and reiterates the importance of the aDSM. Mental well-being, including early identification of depression and psychosocial support should be integral part of routine TB care.

## Supplementary Material

Supplementary material

## Figures and Tables

**Table 1.** Characteristics of SAE incidence and time to occurrence by type among DR-TB patients in Georgia that initiated treatment between 2016 and 2018 (N=73).

SAE Type	Grade of SAE					Total n, %	Number (%) of patients with SAE	Rate per 100 P-M	Median time to SAE (days)	IQR (days)	Earliest onset (days)	Latest onset (days)
	I	II	III	IV	NA							
Cardiotoxicity	2	–	3	5	–	10 (13.7)	9 (2.3)	0.16	124	77–230	8	367
Hepatotoxicity	–	1	12	3	–	16 (21.9)	14 (3.6)	0.26	194	103–289	42	560
Nephrotoxicity	2	–	3	–	–	5 (6.8)	5 (1.3)	0.08	377	195–457	38	466
Neurotoxicity	–	–	3	1	1	5 (6.8)	5 (1.3)	0.08	110	69–325	17	381
Electrolytes disbalance	–	–	–	–	–	0 (0)	0 (0)	0	–	–	–	–
Gastrointestinal disorders	2	3	–	–	1	6 (8.2)	5 (1.3)	0.10	197	64–404	46	493
Other	2	–	13	7	9	31 (42.5)	23 (6.8)	0.49	190	308	5	700
Total	8	4	34	16	11	73 (100)	49 (12.6)	1.16	183	84–334	5	700

SAE, serious adverse event; P-M, person month; IQR, interquartile range; NA, not available.

**Table 2.** SAE characteristics by type outcome, actions taken and suspected anti-TB medication for DR-TB patients in Georgia initiating treatment between 2016 and 2018 (N=73).

Characteristics	Total, n (%)	Cardio-toxicity, n (%)	Hepato-toxicity, n (%)	Nephro-toxicity, n (%)	Neuro-toxicity, n (%)	Gastro-intestinal n (%)	Other, disorders, n (%)
Total	73	10	16	5	5	6	31
**Outcome**							
Resolved	44 (60.3)	9 (90.0)	10 (62.5)	4 (80.0)	3 (60.0)	4 (66.7)	14 (45.2)
Not resolved	13 (17.8)	–	4 (25.0)	–	1 (20.0)	–	8 (25.8)
Resolved with sequelae	6 (8.2)	1 (10.0)	2 (12.5)	1 (20.0)	1 (20.0)	–	1 (3.2)
Resolving	3 (4.1)	–	–	–	–	2 (33.3)	1 (3.2)
Fatal	7 (9.6)	–	–	–	–	–	7 (22.6)
**Actions taken**							
Dose maintained	11 (15.1)	2 (20.0)	–	1 (20.0)	–	1 (16.7)	7 (22.6)
Dose reduced	1 (1.4)	–	–	–	1 (20.0)	–	–
Drug permanently withdrawn	3 (4.1)	–	2 (12.5)	–	1 (20.0)	–	–
Drug interrupted	39 (53.4)	7 (70.0)	12 (75.0)	3 (60.0)	1 (20.0)	3 (50.0)	13 (41.9)
Not applicable	13 (17.8)	–	2 (12.5)	1 (20.0)	–	–	10 (32.3)
Not recorded	6 (8.2)	1 (10.0)	–	–	2 (40.0)	2 (33.3)	1 (3.2)
**Suspected anti-TB medication** *							
Bedaquiline	11 (15.1)	4 (60.0)	2 (12.5)	–	1 (20.0)	2 (33.3)	2 (6.5)
Delamanid	15 (20.5)	5 (50.0)	3 (18.8)	–	–	2 (33.3)	5 (16.1)
Linezolid	13 (17.8)	–	5 (31.2)	–	3 (60.0)	1 (16.6)	4 (12.9)
Clofazimine	21 (28.8)	7 (70.0)	9 (56.2)	–	1 (20.0)	2 (33.3)	2 (6.5)
Imipenem/Cilastatin	2 (2.9)	–	1 (6.2)	–	–	1 (16.6)	–
Moxifloxacin	–	–	–	–	–	–	–
Levofloxacin	–	–	–	–	–	–	–
Cycloserine	13 (17.8)	–	4 (25.0)	–	3 (60.0)	1 (16.6)	5 (16.1)
At least one of them	36 (49.3)	8 (80.0)	10 (62.5)	0 (0)	4 (80.0)	5 (83.3)	9 (29.0)

* Multiple drugs could be suspected. Sum of column percentages may exceed 100%. RR, rifampicin resistance; SAE, serious adverse event; TB, tuberculosis.

**Table 3.** Factors associated with SAE development among DR-TB patients in Georgia that initiated treatment between 2016 and 2018 (N=388).

		SAE	PM	SAE rate (per 100 PM)	HR	95%CI	p-value	aHR	95%CI	p-value
Sex	Male	59	4802	1.23	1.20	0.68–2.12	0.526	0.93	0.46–1.88	0.840
	Female	14	1495	0.94		ref.			ref.	
Age, years	<29	10	1702	0.59	0.52	0.26–1.03	0.057	0.53	0.22–1.23	0.139
	30–39	20	1637	1.22	0.81	0.45–1.46	0.487	0.87	0.42–1.79	0.706
	40–49	21	1349	1.56	1.07	0.59–1.94	0.832	1.03	0.52–2.07	0.934
	>50	22	1609	1.37		ref.			ref.	
BMI	<18	11	1103	1.00	0.84	0.43–1.62	0.594	0.86	0.43–1.72	0.669
	18–24	55	4016	1.37		ref.			ref.	
	24>	7	1178	0.59	0.57	0.27–1.24	0.157	0.49	0.21–1.12	0.091
Case	New	28	3038	0.92		ref.			ref.	
	Retreated	45	3259	1.38	1.31	0.82–2.11	0.257	1.15	0.69–1.90	0.596
HIV status	Positive	7	371	1.89	1.49	0.67–3.31	0.324	1.68	0.72–3.93	0.229
	Negative	62	5487	1.13		ref.			ref.	
	Not recorded	4	439	0.91	0.79	0.38–1.64	0.520	0.88	0.38–2.06	0.776
HCV	Positive	10	779	1.28	0.92	0.48–1.77	0.796	0.64	0.29–1.44	0.282
	Negative	39	2856	1.37		ref.			ref.	
	Not recorded	24	2662	0.90	0.69	0.41–1.14	0.147	0.63	0.37–1.07	0.089
Diabetes	Yes	8	581	1.38	1.16	0.54–2.47	0.700	1.03	0.40–2.63	0.956
	No	65	5716	1.14		ref.			ref.	
Tobacco use	Yes	38	3123	1.22	1.06	0.67–1.68	0.812	0.75	0.44–1.29	0.301
	No	35	3174	1.10		ref.			ref.	
Alcohol use	Yes	39	2531	1.54	1.52	0.95–2.44	0.084	1.66	0.98–2.81	0.059
	No	34	3766	0.90		ref.			ref.	
ID use	Yes	2	280	0.71	0.81	0.22–3.08	0.763	–	–	–
	No	71	6017	1.18		ref.		–	–	–
Type of resistance	RR/MDR	18	2510	0.72		ref.			ref.	
	pre-XDR	38	3082	1.23	1.62	0.92–2.85	0.093	1.54	0.89–2.67	0.112
	XDR	17	705	2.41	2.77	1.45–5.30	0.002	2.18	1.12–4.23	0.021

SAE, serious adverse event; P-M, person-month; HR, hazard ratio; CI, confidence interval; BMI, body mass index; HIV, human immunodeficiency virus; HCV, hepatitis C; ID, intravenous drug; RR, rifampicin resistance; MDR, multidrug-resistance; XDR, extensive drug resistance.

## References

[1] WHO. Global tuberculosis report 2019 Geneva: World Health Organization; 2019:1–297. Available from: https://www.who.int/tb/publications/global_report/en/

[2] WHO Regional Office for Europe/European Centre for Disease Prevention and Control. Tuberculosis surveillance and monitoring in Europe 2019–2017 data Copenhagen: WHO Regional Office for Europe. 2019.

[3] Diel R, Vandeputte J, De Vries G, Costs of tuberculosis disease in the European Union: A systematic analysis and cost calculation. Eur Respir J 2014;43:554–65. 23949960 10.1183/09031936.00079413

[4] Falzon D, Gandhi N, Migliori GB, Resistance to fluoroquinolones and second-line injectable drugs: Impact on multidrug-resistant TB outcomes. Eur Respir J 2013;42:156–68. 23100499 10.1183/09031936.00134712 PMC4487776

[5] Migliori GB, Tiberi S, Zumla A, MDR/XDR-TB management of patients and contacts: Challenges facing the new decade. The 2020 clinical update by the Global Tuberculosis Network. Int J Infect Dis 2020;92S:S15–25. 32032752 10.1016/j.ijid.2020.01.042

[6] Awofeso N Anti-tuberculosis medication side-effects constitute major factor for poor adherence to tuberculosis treatment. Bull World Health Organ 2008;86:B–D. 10.2471/BLT.07.043802 PMC2647396 18368191

[7] WHO. Active tuberculosis drug-safety monitoring and management Geneva: World Health Organization; 2015. Available from: https://www.who.int/tb/publications/aDSM/en/

[8] Akkerman O, Aleksa A, Alffenaar JW, Surveillance of adverse events in the treatment of drug-resistant tuberculosis: A global feasibility study. Int J Infect Dis 2019;83:72–6. 30953827 10.1016/j.ijid.2019.03.036

[9] Borisov S, Danila E, Maryandyshev A, Surveillance of adverse events in the treatment of drug-resistant tuberculosis: First global report. Eur Respir J 2019;54:1901522. 31601711 10.1183/13993003.01522-2019

[10] WHO. Tuberculosis data Accessed on: 2019 Nov 22. Available from: https://www.who.int/tb/data/en/

[11] Arora S, Kalishman SG, Thornton KA, Project ECHO: A telementoring network model for continuing professional development. J Contin Educ Health Prof 2017;37:239–44. 29189491 10.1097/CEH.0000000000000172

[12] WHO. Companion handbook to the WHO guidelines for the programmatic management of drug-resistant tuberculosis Geneva: World Health Organization; 2015. Available from: https://apps.who.int/iris/bitstream/handle/10665/130918/9789241548809_eng.pdf?sequence=1&isAllowed=y 25320836

[13] U.S. Department of Health and Human Services, National Institutes of Health, National Institute of Allergy and Infectious Diseases. Division of AIDS table for grading the severity of adult and pediatric adverse events. Corrected Version 2.1 Available from: https://rsc.niaid.nih.gov/clinical-research-sites/table-grading-severity-adult-pediatric-adverse-events-changes-highlighted

[14] R Core Team. A Language and Environment for Statistical Computing R Foundation for Statistical Computing; 2018.

[15] Hewison C, Bastard M, Khachatryan N, Is 6 months of bedaquiline enough? Results from the compassionate use of bedaquiline in Armenia and Georgia. Int J Tuberc Lung Dis 2018;22:766–72. 29914602 10.5588/ijtld.17.0840

[16] Mbuagbaw L, Guglielmetti L, Hewison C, Outcomes of bedaquiline treatment in patients with multidrug-resistant tuberculosis. Emerg Infect Dis 2019;25:936–43. 31002070 10.3201/eid2505.181823 PMC6478224

[17] Lorent N, Sebatunzi O, Mukeshimana G, Incidence and risk factors of serious adverse events during antituberculous treatment in Rwanda: A prospective cohort study. PLoS One 2011;6:e19566. 21611117 10.1371/journal.pone.0019566 PMC3097195

[18] Van der Walt M, Lancaster J, Odendaal R, Serious treatment related adverse drug reactions amongst anti-retroviral naïve MDR-TB patients. PLoS One 2013;8:e58817. 23573193 10.1371/journal.pone.0058817 PMC3615995

[19] Schnippel K, Firnhaber C, Berhanu R, Adverse drug reactions during drug-resistant TB treatment in high HIV prevalence settings: a systematic review and meta-analysis. J Antimicrob Chemother 2017;72:1871–9. 28419314 10.1093/jac/dkx107

[20] Borisov SE, Dheda K, Enwerem M, Effectiveness and safety of bedaquiline-containing regimens in the treatment of MDR- and XDR-TB: a multicentre study. Eur Respir J 2017;49:1700387. 28529205 10.1183/13993003.00387-2017

[21] Kalandarova L, Tillashaikhov M, Parpieva N, Treatment outcomes and adverse reactions in patients with multidrug-resistant tuberculosis managed by ambulatory or hospitalized care from 2010–2011 in Tashkent, Uzbekistan. Public Heal Panor 2016;02:21–9.

[22] WHO. WHO consolidated guidelines on tuberculosis. Module 4: treatment - drug-resistant tuberculosis treatment Geneva: World Health Organization; 2020. Available from: https://apps.who.int/iris/handle/10665/332397 32603040

[23] Hwang TJ, Wares DF, Jafarov A, Safety of cycloserine and terizidone for the treatment of drug-resistant tuberculosis: a meta-analysis. Int J Tuberc Lung Dis 2013;17:1257–66. 23735593 10.5588/ijtld.12.0863

[24] Walker IF, Khanal S, Hicks JP, Implementation of a psychosocial support package for people receiving treatment for multidrug-resistant tuberculosis in Nepal: A feasibility and acceptability study. PLoS One 2018;13:e0201163. 30048495 10.1371/journal.pone.0201163 PMC6062069

[25] Huque R, Elsey H, Fieroze F, “Death is a better option than being treated like this”: a prevalence survey and qualitative study of depression among multi-drug resistant tuberculosis in-patients. BMC Public Health 2020;20:848. 32493337 10.1186/s12889-020-08986-x PMC7268321

