# Supplementary material for: Incidence rate and time to serious adverse events among rifampicin resistant tuberculosis patients in Georgia treated with new and repurposed anti-tuberculosis drugs, 2016–2018
Source: Monaldi Arch Chest Dis. Author manuscript; Available in PMC 2022 Aug 23. (PMC9396672; doi:10.4081/monaldi.2021.1649)
Supplement: Supplementary material [file NIHMS1827066-supplement-Supplementary_material.pdf]

SUPPLEMENTARY MATERIAL

DOI: 10.4081/monaldi.2021.1649

Incidence rate and time to serious adverse events among rifampicin resistant tuberculosis patients in Georgia treated with new and repurposed anti-tuberculosis drugs, 2016-2018

Mariana Buziashvili[1], Hayk Davtyan[2], Yuliia Sereda[3], Olga Denisiuk[4], Ogtay Gozalov[3], Nino Lomtadze[1], Arax Hovhannesyan[3]

[1]Scientific Research Unit, National Center for Tuberculosis and Lung Diseases, Tbilisi, Georgia; [2]Tuberculosis Research and Prevention Center, Yerevan, Armenia; [3]World Health Organization, Regional Office for Europe, Copenhagen, Denmark; [4]Alliance for Public Health, Kyiv, Ukraine

Corresponding Author: Mariana Buziashvili, National Center for Tuberculosis and Lung Diseases, 8 Achara str, Tbilisi 0101, Georgia. Tel. +995.597.505098. E-mail: buziashvili.mari@gmail.com

Key words: Tuberculosis; drug resistance; new/repurposed anti-TB drugs; SAEs.

Creative Commons Attribution SNGO

Supplementary Table 1. Definition of terms.

RR-TB	Resistance to rifampicin without resistance to any other anti-TB medications, including fluoroquinolones and second-line injectable agents.
MDR-TB	Resistance to additional anti-TB medications other than rifampicin, without resistance to any of fluoroquinolones and second-line injectable agents.
Pre- XDR-TB	MDR-TB with additional resistance to any fluoroquinolone or at least one of 3 injectable second-line drugs but not both of the categories.
XDR-TB	MDR-TB with additional resistance to any fluoroquinolone and at least one of 3 injectable second-line drugs.
SAE	Any untoward occurrence in a patient given a pharmaceutical product that result in death, are life threatening, requires hospitalization, or prolongation of hospitalization, results in persistent or significant disability/incapacity, causes congenital abnormality/birth defect, is considered otherwise medically important.
Cardiotoxicity	Any kind of drug-induced/worsened toxicity to cardiovascular system, diagnosed by a cardiologist on a basis of relevant tests.
Hepatotoxicity	Any kind of drug-induced/worsened toxicity to liver, diagnosed by presence of relevant symptoms and liver enzymes tests.
Nephrotoxicity	Any kind of drug-induced/worsened toxicity to renal function, based on relevant tests of creatinine and potassium.
Neurotoxicity	Any kind of drug-induced/worsened alteration to the normal activity of the nervous system, diagnosed by neuropathologist's evaluation.
Gastrointestinal disorders	Any kind of drug-induced/worsened toxicity to gastrointestinal system based on presence/severity of relevant symptoms.

RR-TB, rifampicin resistant tuberculosis; MDR-TB, multi-drug resistant tuberculosis; XDR-TB, extensively drug-resistant tuberculosis.

Creative Commons Attribution 3.0

Supplementary Table 2. Characteristics of DR-TB patients in Georgia initiated treatment between 2016 and 2018 (N=388).

Characteristics		n	%
Sex	male	304	78.3
	female	84	21.6
Age, years	≤29	103	26.6
	30-39	102	26.3
	40-49	87	22.4
	≥50	96	24.7
	mean (SD)	39.6 (13.1)	
BMI	<18	70	18.0
	18-24	250	64.4
	>24	68	17.5
	Median (25th-75th percentiles)	20.2 (18.4-23.2)	
Case	new	174	44.9
	previously treated	214	55.1
HIV status	positive	26	6.7
	negative	333	85.8
	not recorded	29	7.5
HCV	yes	57	14.7
	no	168	43.3
	not recorded	163	42.0
Diabetes	yes	33	8.5
	no	355	91.5
Tobacco use	yes	206	53.1
	no/not recorded	182	46.9
Alcohol use	yes	156	40.2
	no/not recorded	232	59.8
Intravenous drug use	yes	21	5.4
	no/not recorded	367	94.6
Type of resistance	RR	4	1.0
	MDR	149	38.4
	pre-XDR	193	49.7
	XDR	42	10.8
Treatment outcome	Cured	212	54.6
	Completed	32	8.2
	Failure	29	7.5
	Death	7	1.8
	Lost to follow up	42	10.8
	Not evaluated	66	17.0

BMI, body mass index; DR, drug-resistant; HIV, human immunodeficiency virus; HCV, hepatitis C; RR, rifampicin resistance; MDR, multidrug-resistance; TB, tuberculosis; XDR, extensively drug resistance.

Creative Commons Attribution 3.0 IGO

Supplementary Table 3. Description of SAEs with fatal outcomes.

SAE with fatal outcome	Possibly related to anti-TB treatment	Suspected drug/regimen	Other reported causal factors
Suicide	Possible	Cs	HCV co-infection, psychotropic drug abuse history
Death	Yes	Cfz, Mfx	Low potassium, increased creatinine, prolonged QT interval
Suicide	Possible	Cs	HCV co-infection, history of depression and anxiety
Sudden death	Yes	Cfz, Dlm	Extremely low BMI, extensive TB disease
Death	No	-	History of cardiac complications
Death	No	-	Metastatic myeloma
Death	No	-	Severe COPD

Creative Commons Attribution 3.0

pagepress
